# Fat shapes fate: unlock the destiny of a cell with single-cell metabolomics

**DOI:** 10.1093/lifemedi/lnac026

**Published:** 2022-08-01

**Authors:** Ziyi Wang, Fei Sun, Wei Xiong

**Affiliations:** Institute on Aging and Brain Disorders, The First Affiliated Hospital of USTC, Division of Life Sciences and Medicine, University of Science and Technology of China, Hefei 230026, China; Department of Cell Biology, Duke University Medical Center, Durham, NC 27710, USA; Institute on Aging and Brain Disorders, The First Affiliated Hospital of USTC, Division of Life Sciences and Medicine, University of Science and Technology of China, Hefei 230026, China; Anhui Province Key Laboratory of Biomedical Aging Research, Hefei 230026, China

Cellular heterogeneity plays a key role in different biological and pathophysiological processes, including development, differentiation, aging, and cancer. With the advance in single-cell technologies, it has been vastly studied and reported at the genomic, epigenetic, transcriptional, and protein levels in various cell populations and tissues. At the cellular level, the differences in cell fate decisions directly reflect cellular heterogeneity. Cell fate is mainly determined by two types of signals: the extracellular signal and the intracellular signal. Many studies focused on extracellular signals such as cytokines, growth factors, and hormones; however, the effect of intracellular signals, such as gene expressions and metabolisms, on cell fate decisions remains to be explored [1].

Lipids are crucial components of organisms, participating in plasma membrane formation, energy metabolism, and signal transduction. They also interact with proteins, altering the function, and distribution of proteins. Eukaryotes possess thousands of types of lipids and each of which has a unique structure and function. The development of lipidomics enables us to study the complexity and the dynamism of lipids more comprehensively. Lipid heterogeneity is discovered in various cell populations, but the roles of lipids in cellular fate determination are unknown. Fibroblasts are a vastly heterogeneous population with the plasticity to transform between different states [2, 3]. The composition of fibroblast subtypes affects the progression of fibrosis and the construction of microenvironments [4, 5]. However, the molecular mechanism underlying fibroblast heterogeneity is still unclear.

**Figure 1.**
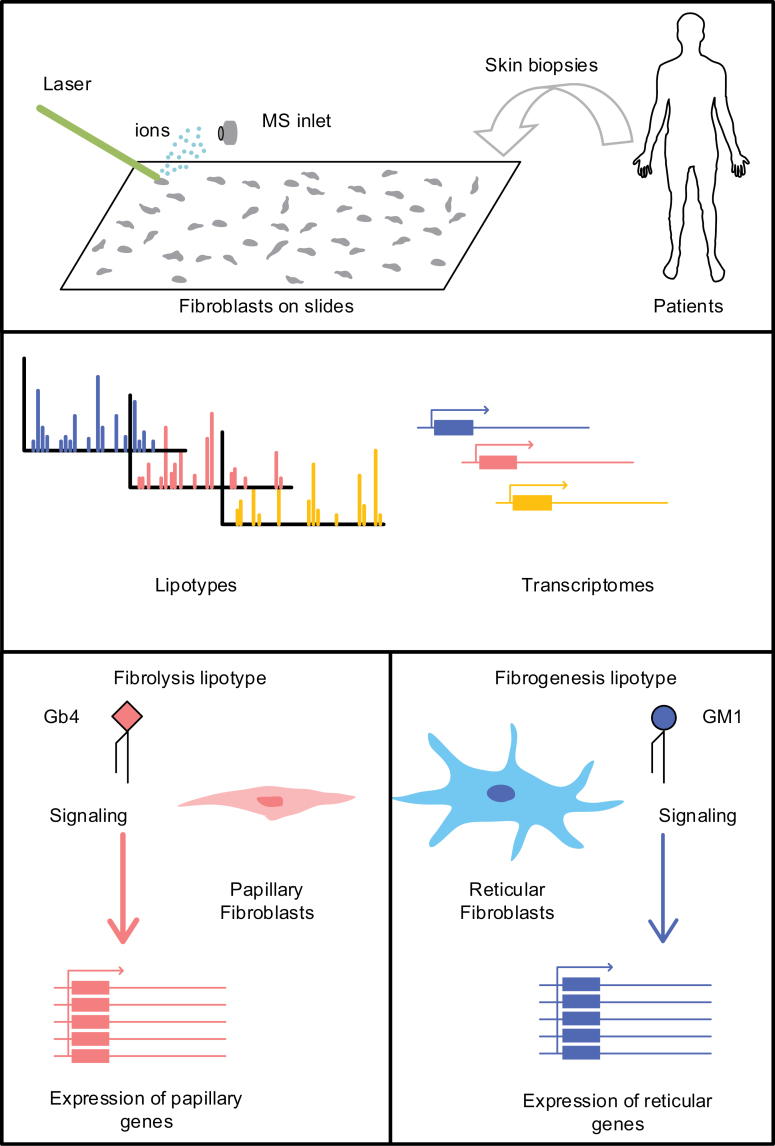
The unexpected relationship between the lipid profile and transcriptome of single fibroblasts determines cell fate and heterogeneity.

Recently, Giovanni D’Angelo’s and Gioele La Manno’s groups revealed the contribution of lipid metabolism in mediating fibroblast fates during dermis development, and sphingolipids are identified as the key factor determining fibroblast heterogeneity [6] (Fig. 1). Their study investigated the lipidome and transcriptome of human dermal fibroblasts at the single-cell level with high-resolution mass spectrometry imaging and scRNA-seq. Researchers found that the lipid composition and transcriptomic states differ from cell to cell. Ranking individual lipids according to their coefficient of variance, sphingolipids are among the tops. Further coregulation analysis reveals that sphingolipids are correlated with other lipids in the lipidome. Changes in sphingolipids have profound influences on the lipidome of a cell. In addition, sphingolipids are markers of fibroblast lipid composition, which regulates cellular responses to extracellular signals. Alterations in lipotypes, the lipid composition of a cell, drive the cells into other states to execute different functions. Lineage-related cells possess similar sphingolipid profiles. These findings suggest that sphingolipid profiles define the phenotypic states of different fibroblasts subtypes.

The lipotype is found to be associated with the transcriptomic features of fibroblasts. Single-cell RNA sequencing reveals that fibroblast transcriptomic heterogeneity corresponds to their lipidomic heterogeneity. This correlation is validated in primary dermal human fibroblasts by co-staining fibroblast subtype markers with sphingolipid-binding toxins. The *in vivo* study with human skin biopsies found that ChTxB+ (Cholera Toxin Subunit B, binding to sphingolipid head group GM1) cells were mainly located in the reticular dermal region with high fibrogenic activities. In contrast, ShTxB1a+/2e+ (Shiga toxin 1a/2e, binding to sphingolipid head group Gb3/4) cells were primarily found in the papillary dermal region with fibrolytic activities. Employing cutaneous squamous cell carcinoma tissue, researchers further demonstrated that cancer-associated fibroblasts located around cancer lesions are predominantly ChTxB+. Overall, these findings suggest that lipotypes mark different fibroblast states *in vivo*.

Mechanistically, this study revealed the interaction between lipids and signaling pathways that mediates the metabolism and gene expressions in fibroblasts. Researchers reported that the FGF2 signaling pathway is modulated by sphingolipids. While globo-series sphingolipids positively regulate FGF2 signals, ganglio-series glycosphingolipids act negatively.

In conclusion, this study provides a comprehensive view of how metabolism modulates cell fate decisions. Specifically, the authors reveal the key role of lipotype in mediating cellular states and fates. Prior to this study, metabolomics was mainly performed with homogenates from bulk tissues or cells. However, cells differ from one another in their metabolic status. While cellular heterogeneity has been studied with single-cell transcriptomic, epigenomic, and proteomic technologies, it has seldomly been investigated at the metabolic level. Using the high-resolution mass spectrometry imaging method, the authors detailly analyzed the single-cell metabolic profile and revealed the lipid metabolic heterogeneity in fibroblasts. This study took the study of metabolomes to the single-cell level and expanded the use of metabolomics in biological research.

Combining single-cell transcriptomics with single-cell metabolomics, this study discovered that the features of the lipidome, like lipotypes, are correlated with the transcriptomic features. The fibroblast subtypes defined by gene expression signatures correspond to the ones defined by lipotypes. The authors explored the regulatory roles of lipid metabolism on cellular state determination and further revealed the underlying molecular mechanism. Moreover, this study demonstrates the correlation between metabolome and transcriptome. Although single-cell omics technologies enable the analysis of cellular features at various levels, the relationship between different omics data is still unclear. Cells are composed of interconnected systems. Crosstalks between systems are required to coordinate biological processes. This study provides novel insights into the integration of molecular features and the interactions between biological processes in a single cell.

Lastly, this study provides a new method for medical research and clinical applications. Conventional pathologic diagnosis is based primarily on the morphology of cells and tissues. Concluding from the limited cellular information provided by morphology is sometimes challenging. The metabolome, however, is a direct reflection of cellular function, and metabolic markers are relatively specific and reliable. The newly developed method combining single-cell metabolome and transcriptome in this study enables high-throughput metabolic profiling of extensive clinical samples for establishing a metabolomic database containing metabolic features and pathways of human diseases. Combined with bioinformatics and machine intelligence, it can be further employed for automatic diagnosis and treatment evaluation in clinics. In summary, this novel technology will advance molecular pathology and precision medicine.

Single-cell metabolomics is a rapidly developing field. Emerging technologies bring new possibilities for basic medical sciences and novel insights into mechanistic studies. A new era for medical research is coming, with mechanisms of diseased elucidated at the single-cell or even single-molecule level. A patch clamp-based single-cell metabolomics approach has been recently developed to provide a real-time, label-free, *in situ* analysis of cells and tissues [7]. The technique is capable of detecting various metabolites, including amino acids, sugars, lipids, and neurotransmitters. Employing this novel technical platform, researchers discovered a novel glutamate biosynthesis pathway that affects brain functions [8]. Glutamate is an important neurotransmitter, playing a crucial role in memory and learning. UV exposure elevates the urocanic acid, the glutamate precursor, in the blood, which traffic to the brain for glutamate synthesis in neurons. This study elucidates the mechanism underlying the beneficial effect of sunlight exposure on learning and memory. It further suggests a novel clinical intervention for neurological diseases such as Alzheimer’s and dementia.

Capillary electrophoresis is another brand-new technology for single-cell metabolomics [9]. It is capable of consecutively capturing the metabolomic information of a live cell in real-time with microcapillaries and acquiring the dynamism of metabolomes during tissue development.

A new field of research emerged recently, focusing on heterogeneity inside the cell. The metabolic networks are highly compartmentalized, with specific enzymes distributing unevenly in a cell. Using gas cluster ion beam–secondary ion mass spectrometry, Pareek *et al.* reveal this cytoplasmic heterogeneity [10]. They demonstrated the metabolic channeling between enzymes in frozen HeLa cells, broadening our understanding of tumor metabolism.

In summary, the development of single-cell metabolomics will uncover the undermining roles of cell metabolism in regulating biological processes. The application of metabolic profiling in clinical research will undoubtedly benefit human health.
